# Structural and magnetic properties of multi-core nanoparticles analysed using a generalised numerical inversion method

**DOI:** 10.1038/srep45990

**Published:** 2017-04-11

**Authors:** P. Bender, L. K. Bogart, O. Posth, W. Szczerba, S. E. Rogers, A. Castro, L. Nilsson, L. J. Zeng, A. Sugunan, J. Sommertune, A. Fornara, D. González-Alonso, L. Fernández Barquín, C. Johansson

**Affiliations:** 1 Department CITIMAC, Faculty of Science, University of Cantabria, 39005 Santander, Spain; 2 Healthcare Biomagnetics Laboratory, University College London, 21 Albemarle Street, London, W1S 4BS, UK; 3 Physikalisch-Technische Bundesanstalt, Abbestr. 2-12, 10587 Berlin, Germany; 4 Bundesanstalt für Materialforschung und –prüfung (BAM), Unter den Eichen 87, 12205 Berlin, Germany; 5 Academic Centre for Materials and Nanotechnology, AGH University of Science and Technology, al. A. Mickiewicza 30, 30-059 Krakow, Poland; 6 ISIS-STFC Neutron Scattering Facility, Harwell Science and Innovation Campus, Didcot, OXON, OX11 0QX, UK; 7 SOLVE Research and Consultancy AB, Lund, Sweden; 8 Lund Centre for Field-Flow Fractionation, Department of Food Technology, Engineering and Nutrition, Lund University, Sweden; 9 Department of Physics, Chalmers University of Technology, 41296 Göteborg, Sweden; 10 SP Technical Research Institute of Sweden, Chemistry, Materials and Surfaces Unit, 11486 Stockholm, Sweden; 11 RISE Acreo, 40014 Göteborg, Sweden

## Abstract

The structural and magnetic properties of magnetic multi-core particles were determined by numerical inversion of small angle scattering and isothermal magnetisation data. The investigated particles consist of iron oxide nanoparticle cores (9 nm) embedded in poly(styrene) spheres (160 nm). A thorough physical characterisation of the particles included transmission electron microscopy, X-ray diffraction and asymmetrical flow field-flow fractionation. Their structure was ultimately disclosed by an indirect Fourier transform of static light scattering, small angle X-ray scattering and small angle neutron scattering data of the colloidal dispersion. The extracted pair distance distribution functions clearly indicated that the cores were mostly accumulated in the outer surface layers of the poly(styrene) spheres. To investigate the magnetic properties, the isothermal magnetisation curves of the multi-core particles (immobilised and dispersed in water) were analysed. The study stands out by applying the same numerical approach to extract the apparent moment distributions of the particles as for the indirect Fourier transform. It could be shown that the main peak of the apparent moment distributions correlated to the expected intrinsic moment distribution of the cores. Additional peaks were observed which signaled deviations of the isothermal magnetisation behavior from the non-interacting case, indicating weak dipolar interactions.

Biomedical applications of iron oxide nanoparticles have attracted considerable interest in the last decades, as summarised by the numerous recent review articles^1^,^2^,^3^,^4^. Of the many proposed uses of iron oxide nanoparticles the most promising examples are cancer treatment by magnetic hyperthermia^5^,^6^,^7^,^8^,^9^,^10^,^11^, magnetic resonance^12^,^13^ and particle imaging^14^,^15^, magnetic biosensing^16^,^17^ as well as magnetic drug targeting^11^,^12^,^13^. The required magnetic response of the nanoparticles is typically determined by the application, and is in turn intrinsically dependent on the structural properties of the particle system. For individual particles the theoretical framework regarding the correlations between the structural properties, such as size and shape, and magnetic properties, such as magnetic moment^18^,^19^ and relaxation times^20^,^21^, is well established.

A classic example of the delicate interplay between magnetic behaviour and structure in nano-particulate systems is that of dilute ensembles of superparamagnetic particles. Here, the size distribution *p(V*) can be directly translated into a moment distribution *p(μ*), which feeds into the Langevin expression to model the isothermal magnetisation behaviour *M(H*)^22^,^23^,^24^. Whilst this model offers an adequate description for *single*-core nanoparticle systems under consideration of polydispersity, recent work has suggested that so called *multi*-core particles^25^,^26^,^27^ may be more suited to magnetic hyperthermia. Refs 5, 6, 7, 8, 9, 10 each observe an increase in magnetic heating for multi-core particles compared to single-cores under the same conditions. Multi-core particles consist of several magnetic cores per particle and consequently, long range dipolar interactions within one multi-core particle can be present. Both experimental studies^28^,^29^,^30^ and simulations^31^,^32^,^33^,^34^ indicate that the presence of magnetic interactions between particles significantly distort the observed magnetisation behaviour of the ensemble.

In the literature, there are several approaches to include dipolar interactions into the classical Langevin framework, which enable to analyse the magnetisation data of coupled nanoparticle ensembles^28^,^29^,^30^,^32^. In the current work we applied an alternative approach. The magnetisation curves of a magnetic nanoparticle ensemble were numerically inversed in order to extract the *apparent* moment distribution, using simply the classical Langevin function as model function. The working hypothesis was that in case of dipolar interactions the extracted *apparent* moment distribution exhibits characteristic distortions compared to the *intrinsic* moment distribution of the nanoparticle ensemble. Similar approaches can be found in refs 35, 36, 37, where different numerical methods were used to determine the discrete moment distribution of nanoparticle ensembles. This study stands out in so far that the numerical approach to extract the moment distribution is identical to the numerical inversion method used to analyse the scattering data.

In this paper the structural and magnetic properties of multi-core particles were determined which consist of iron oxide nanoparticle cores embedded in poly(styrene) spheres and which have been established as promising candidates for a wide range of biomedical applications^27^. The main goals of this study were to disclose the arrangement of the cores within the poly(styrene) spheres and to investigate the influence of dipolar interactions on their magnetisation behaviour. For this purpose a combination of static light scattering (SLS), small angle X-ray scattering (SAXS), small angle neutron scattering (SANS) and quasi-static DC magnetisation measurements was applied. Additionally, the core size and hydrodynamic size of the multi-core particles were determined by transmission electron microscopy (TEM), X-ray diffraction (XRD) and asymmetrical flow field-flow fractionation in combination with multi-angle light scattering (AF4-MALS).

To determine the structural arrangement of the cores the SLS, SAXS and SANS experiments of the particles dispersed in water were analysed with a model-independent approach^38^,^39^. Here initially the real space function *P(r*) is determined by an indirect Fourier transform of the reciprocal scattering data *I(q*)^38^,^39^,^40^,^41^. The indirect Fourier transform is simply a numerical inversion of the data and no a priori assumptions have to be done regarding the particle shape, in contrast to classical model fits^42^. The extracted function *P(r*) is the so-called pair distance distribution function, which has characteristic shapes depending on the geometry of the scatterers^41^. Physical data analysis is ultimately performed by comparing the extracted pair distance distribution functions with the distribution functions of particles with known geometries. This approach is often used for the structural analysis of complex biological systems – such as micellar^42^,^43^,^44^ or polymer solutions^41^,^45^.

The same numerical approach as for the indirect Fourier transform was also applied to isothermal magnetisation measurements to extract the apparent moment distributions *p(μ*). As model function simply the Langevin function was used and data analysis was ultimately performed by comparing the extracted apparent distributions *p(μ*) with the expected intrinsic moment distributions of the cores. As mentioned above, the working hypothesis was that for example in case of dipolar interactions, the apparent moment distributions exhibit characteristic distortions. Hence, this is an alternative approach compared to established model fits^28^,^29^,^30^,^32^ where the influence of dipolar interactions is a priori included in the model functions.

This work was performed within the European FP7 project NanoMag^46^ which aims to implement a roadmap for the standardisation of the characterisation of magnetic nanoparticles and redefine existing analysis methods^47^. We show that the numerical method presented here provides a valid approach with which to determine the intrinsic properties of any nanoparticle system.

## Sample

### Synthesis

The synthesis of the multi-core particles used in this study is described in detail in refs 25 and 27. Briefly, the sample comprises iron oxide nanoparticle cores embedded in poly(styrene) spheres. The multi-core particles were prepared by a controlled precipitation process of the polymer which traps the cores in emulsion droplets *via* solvent evaporation^27^. For the majority of the characterisation techniques, the particles were colloidally dispersed in water with an iron content of *c*_*Fe*_ = 6.9 mg_Fe_/ml.

### Basic physical characterisation

We begin the discussion by first comparing the results of TEM, XRD and AF4-MALS. Together, these techniques provide a visualisation of the particle system and the ensemble.

**Figure 1:**
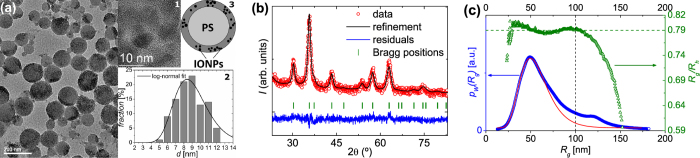
Results of the basic physical characterisation of the multi-core particles via TEM, XRD and AF-MALS. (**a**) TEM image of the multi-core particles. *Inset 1*: High resolution image of the iron oxide nanoparticle cores. *Inset 2*: Histogram of measured diameters of the cores and fit with a log-normal function (〈*D*〉 = 9.0 nm, *σ* = 0.23). *Inset 3*: Sketch of the envisioned sphere-shell particle structure of the spherical poly(styrene) particles with the embedded iron oxide nanoparticle cores as the shell. (**b**) Rietveld refinement of the X-ray diffraction pattern obtained at 300 K, including the residuals. Vertical tick marks indicate the position of the allowed diffraction peaks. (**c**) Weight-based particle size distribution *p*_*W*_ (blue circles) and ratio of gyration and hydrodynamic radius (shape parameter) *R*_*g*_/*R*_*h*_ (green triangles) as a function of *R*_*g*_ determined by AF4-MALS. Red line: Distribution 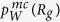 determined by fitting first peak of *p*_*W*_(*R*_*g*_) with a log-normal distribution.

The size and shape of both individual cores and multi-core particles were primarily analysed by TEM (Fig. 1(a)). The diameter of 200 cores was measured and combined to form a frequency distribution (inset, Fig. 1(a)). The core diameters are at first approximation log-normally distributed, with the best fit yielding a mean core diameter of 〈*D*〉 = 9.0(2) nm. The core size was additionally determined by analysing the XRD pattern of the freeze-dried powder, using Rietveld analysis^48^. The Rietveld refinement of the scattering pattern shown in Fig. 1(b) was obtained by assuming a *Fd* 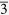
*m* space group. The lattice parameter was determined to be 8.355, which lies between the values of bulk maghemite (8.336) and magnetite (8.397)^18^ and indicates that the iron oxide cores are non-stoichiometric mixtures of maghemite/magnetite. The best fit of the data indicates a mean core size of 7(1)nm, which is slightly less than that observed by TEM.

TEM also provides insight into the total diameter of the multi-core particles. We observe that the particle diameter is polydisperse and lies in the range ~100–150 nm. The hydrodynamic size distribution of the multi-core particles was determined by AF4-MALS^49^,^50^.

The two key parameters determined by AF4-MALS that are of most use to this study are the weight-based size distribution and the shape parameter *R*_*g*_/*R*_*h*_ (Fig. 1(c)), where *R*_*g*_ is the radius of gyration and *R*_*h*_ the hydrodynamic radius. Figure 1(c) shows that the shape parameter is essentially constant with a value of *R*_*g*_/*R*_*h*_ ≈ 0.79 for *R*_*g*_ < 100 nm. For *R*_*g*_ < 100 nm, however, this ratio *R*_*g*_/*R*_*h*_ rapidly decreases, which implies that particles with gyration radius larger than 100 nm significantly deviate from the geometry of the primary particles. We surmise that particles with *R*_*g*_ > 100 nm are actually agglomerates of the individual particles.

We observe on distinct peak in the region up to 100 nm in the weight-based size distribution *p*_*W*_(*R*_*g*_) with its maximum at *R*_*g*_ = 49 nm. Assuming that this peak arises from individual particles, we can gain further insight by modeling with a log-normal size distribution The best fit result, shown in Fig. 1(c), indicates a mean radius of 〈*R*_*g*_〉 = 57 nm. Quantification of the shape parameter allows us to calculate both the mean and maximum value of the hydrodynamic diameter of the particles. With *R*_*g*_/*R*_*h*_ ≈ 0.79 the mean hydrodynamic size 〈*D*_*h*_〉 ≈ 2 ⋅ 57 nm/0.79 = 144 nm and the maximal value *D*_*h,max*_ ≈ 2 ⋅ 100 nm/0.79 = 253 nm. The hydrodynamic diameter, however, is typically larger than the physical size due to the presence of solvation layers^51^, and thus these values should be regarded as upper limits of the particle sizes.

Finally, we note that the value of the shape parameter (0.79) confirms our assertion that the cores are distributed in the outer surface of the polymer spheres, as schematically shown in Fig. 1(a). For a sphere with radius *R* and a homogeneous scattering length density, the gyration radius is related to the physical radius by 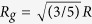. However, in the extreme case of a hollow sphere with mass distributed within an infinitesimally thin shell, the ratio *R*_*g*_/*R* approaches 1^52^. The particles measured here have a value that is systematically greater than 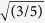. This indicates that the density in the outer shell of the spherical multi-core particles is increased, which makes sense physically as the scattering length density of iron oxide is larger than that of poly(styrene). We have tested this assumption by analysing the small angle scattering behaviour of the particles, described below.

## Structural properties

Here, we describe the characterisation of the structural properties of the nanoparticles, which have been ascertained using a model independent approach to analyse both light, X-ray and neutron scattering data. It is pertinent to begin this discussion by briefly describing the theoretical basis of the scattering techniques. Our approach is firstly illustrated by applying the indirect Fourier transformation (IFT) to an ideal model system to yield the pair distance distribution functions and subsequently to the experimentally observed data. Further details on the numerical approach used to analyse the data can be found in the Methods section.

### Theory

With SLS, SANS and SAXS the angular distribution of the time-averaged scattering intensity *I* as a function of the scattering wave vector *q* = (4*π*/*λ*)sinΘ is measured. Here, 2Θ is the scattering angle and *λ* the wavelength of light (SLS), X-rays (SAXS) or neutrons (SANS). The wavelength of visible light used for SLS is in the order of 600 nm whereas for SAXS and SANS *λ* ≈ 0.1 − 1 nm. Hence, with SLS the scattering intensity *I(q*) is measured in a much lower *q*-range compared to both SAXS and SANS, and so which enables the characterisation of larger scatterers.

The scattering intensity in reciprocal space, *I(q*), is related to the real space function *P(r*) in the following way:


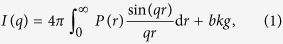


where *bkg* is the *q*-independent background due to incoherent scattering contributions^53^. This real space function *P(r*) is the so-called pair distance distribution function (PDDF). For a particle with arbitrary shape, this PDDF can be written^54^


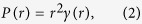


where *γ(r*) is the convolution square of the scattering length density (SLD) contrast Δ*ρ(r*) averaged over all directions in space.

For a particle with a homogeneous scattering length density *ρ*_*p*_, and which is dispersed in a matrix with *ρ*_*m*_, the absolute value for the contrast of the scattering length density is determined by the difference between the particles and the matrix (Δ*ρ* = *ρ*_*p*_ − *ρ*_*m*_), and is ultimately technique dependent. For example, in SLS the difference is proportional to the refractive index difference, whilst for SAXS it is proportional to the difference in electron density contrast (determined by atomic number *Z*)^55^. Neutrons, on the other hand, interact with the nuclei and their scattering is ultimately determined by the strength of the neutron-nucleus interactions.

In a conventional two-phase system, however, the profile of Δ*ρ(r*) - and hence *γ(r*) and *P(r*) - is independent of the applied technique, and instead depends only upon the shape of the scatterer. In the most simple case of a spherical particle with diameter *D*, the PDDF is expressed as:


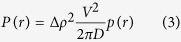


with


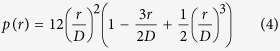


for 0 < *r* < *D* and *P(r*) = 0 for *r* ≥ *D*, as shown in ref. 41. The maximum of *P(r*) is at 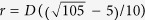.

When characterising an ensemble of nanoparticles, however, one must also consider the inherent polydispersity. In this case, the experimentally detected scattering intensity is proportional to the Fourier transform of the average PDDF with the absolute values dominated by *P(r*) ∝ *D*^5^ i.e. large particles. The real space distribution function is typically extracted from reciprocal space scattering data using an indirect Fourier transform (IFT)^38^,^39^,^40^,^41^. We have used an approach based upon a regularised numerical inversion similar to references^38^,^39^. Precise details of the approach are provided in the Methods section. In the following section we describe the type of information that can be extracted from the pair distance distribution functions.

### Calculated data

AF4-MALS measurements suggest that the multi-core particles consist of poly(styrene) spheres, which were surrounded by a surface layer with embedded iron oxide cores (Fig. 1(a)). In order to simulate physically meaningful PDDFs, we first need to model the neutron and X-ray scattering patterns of these particles. For this purpose we need to make some assumptions regarding the particle geometry.

The scattering intensities *I(q*) were calculated with the form factor of a sphere surrounded by a shell with homogeneous scattering length density^56^. For all scattering experiments the particles were colloidally dispersed in water (solvent). For the sphere, the shell and the solvent the SLDs expected for neutrons and X-rays were used for the calculations, respectively (Table 1).

For neutrons, the SLDs for both poly(styrene) and iron oxide are above that of water but in case of X-rays the SLD of poly(styrene) is below. Hence, in SANS, the particles can be at first approximation represented by particles with *ρ*_*shell*_ > *ρ*_*sphere*_ > *ρ*_*solvent*_. For SAXS, however, the particles constitute particles with *ρ*_*shell*_ > *ρ*_*solvent*_ > *ρ*_*sphere*_, provided the number of iron oxide nanoparticle cores is sufficient to raise locally the SLD in the shell above *ρ*_*solvent*_. For the calculations, we have assumed that the presence of iron oxide nanoparticles either on or at the surface of the poly(styrene) spheres increases the scattering length density moderately and that on average the shell has a homogeneous SLD. For neutrons we assumed for the calculations *ρ*_*shell*_ = 1.8 ⋅ 10^−4^1/nm^2^ and for X-rays *ρ*_*shell*_ = 9.7 ⋅ 10^−4^1/nm^2^ (Table 1).

Regarding the geometry of the particles a diameter of the poly(styrene) sphere of *D*_*s*_ = 120 nm and a shell thickness of *t* = 20 nm was assumed. Thus the total size of the simulated particles was *D*_*max*_ = *D*_*s*_ + 2*t* = 160 nm which is in the range of the mean hydrodynamic size of the multi-core particles, as determined by AF4-MALS. With these assumptions we have been able to model the scattering profiles, to which we added a reasonable standard deviation of *σ(q*) = 0.01 ⋅ *I(q*).

**Figure 2:**
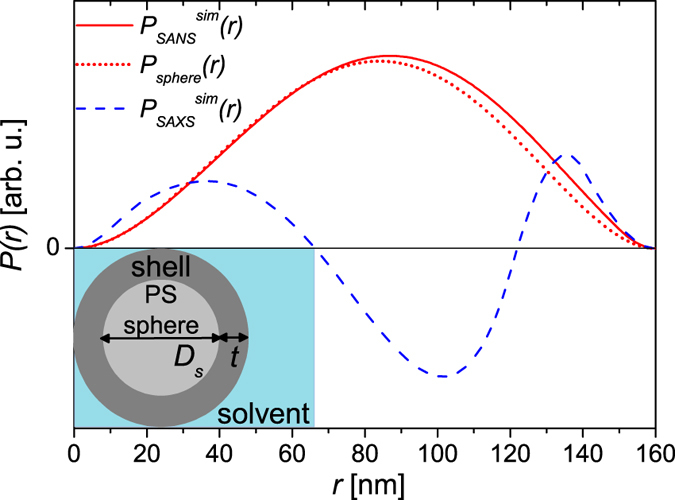
Simulated PDDFs. Comparison of simulated PDDFs for small angle scattering via neutrons (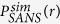) and X-rays (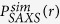) from a 120 **nm poly(styrene) sphere with a 20 nm thick shell embedded in water, as well as the calculated profile of a homogeneous sphere with** *D* = 160 nm (equation 3). Assumed scattering length densities for neutrons and X-rays are provided in Table 1.

At this stage we can now numerically inverse the simulated scattering data *I(q*) (details provided in the Methods section). To test the IFT, the maximum size *D*_*max*_ was varied from 100–200 nm in 2 nm steps although the actual size of the particles was known. The regularisation parameter, *α*, was varied over several orders of magnitude in 70 steps. Figure 2 compares the distribution functions obtained with the highest evidence for SANS (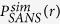) and SAXS (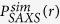).

For both distributions *D*_*max*_ was determined to be 160 nm, which is in agreement with the *real* size of the simulated particles. The profile of both distributions is, however, significantly different due to the different scattering length density distributions used for the scattering of neutrons and X-rays. As can be seen in Fig. 2, the PDDF obtained for neutrons is bell-shaped with one distinct maximum at *r* = 87 nm. The distribution is basically identical to the PDDF *P*_*sphere*_(*r*) calculated for a homogeneous sphere with equation 3 for *D* = 160 nm, where the maximum is at 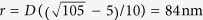. Only in the large *r*-range the values are slightly increased due to the higher scattering length density assumed for the shell.

However, a significantly different distribution is observed for X-rays and this is due to the fact that *ρ*_*sphere*_ < *ρ*_*solvent*_ but *ρ*_*shell*_ > *ρ*_*solvent*_. As a result, the pair distance distribution function exhibits two distinct peaks and takes on negative values in the region *D*_*s*_/2 < *r* < *D*_*s*_.

Comparable particle systems were experimentally analysed e.g in refs 43, 44, 45,57 and simulated in ref. 58. Interestingly, in these cases the authors observed quasi-symmetric functions when the main body of the particle scattered more than the solvent, and functions qualitatively identical to that observed here – with two peaks – when *ρ*_*shell*_ > *ρ*_*solvent*_ > *ρ*_*sphere*_.

We thus note that the PDDFs of particles obtained by an indirect Fourier transform of scattering data directly reveal two important structural parameters: firstly, we obtain the maximum size (*D*_*max*_) of the particles. Secondly, we also gain qualitative insights on the nature of the scattering length density profile, which in turn tells us valuable information about the overall structure of the particles. Having now demonstrated our approach, we apply it to experimentally observed scattering measurements of the particles so as to ascertain their internal structure.

### Experimental data analysis

**Figure 3:**
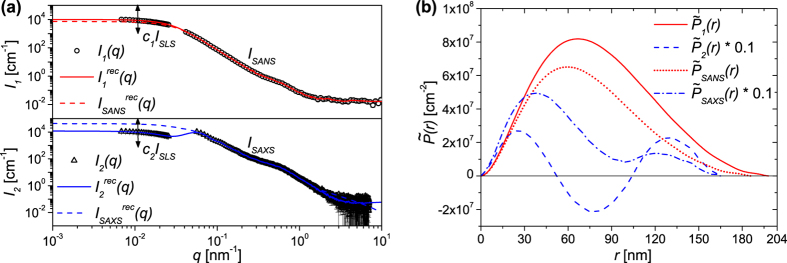
Results of the structural characterisation of the multi-core particles via SANS, SAXS and SLS. (**a**) The experimentally measured scattering intensities *I*_*SANS*_(*q*), *I*_*SAXS*_(*q*) and *I*_*SLS*_(*q*). The static light scattering intensity was scaled by *c*_1_ and *c*_2_, respectively. The reconstructed curves 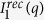, 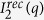, 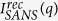 and 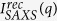 were calculated for the distributions 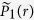, 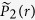, 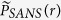 and 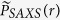 from Fig. 3(b). (**b**) The pair distance distribution functions 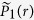, 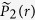, 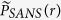 and 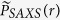 determined by an indirect Fourier transform of *I*_1_(*q*), *I*_2_(*q*), *I*_*SANS*_(*q*) and *I*_*SAXS*_(*q*).

In Fig. 3(a) we compare the scattering intensities from SANS and SAXS. We have combined each of the data sets with the scattering intensity from SLS, so that the total intensity of each method is given by *I*_1_(*q*) = (*c*_1_ ⋅ *I*_*SLS*_(*q*), *I*_*SANS*_(*q*)) and *I*_2_(*q*) = (*c*_2_ ⋅ *I*_*SLS*_(*q*), *I*_*SAXS*_(*q*)), respectively. In doing so we have expanded the *q*-range significantly. Following this, the two combined data sets *I*_1_(*q*) and *I*_2_(*q*) were inversed with the IFT to extract the pair distance distribution functions, as shown in detail in the Methods section. For this purpose, the IFT of *I*_1_(*q*) and *I*_2_(*q*) was performed for 201 different *D*_*max*_ values and 51 different values of the scaling factors *c*_1_ and *c*_2_. Additionally, for each parameter set [*D*_*max*_, *c*_1,2_] the regularisation parameter *α* was varied logarithmically over several orders of magnitude in 70 steps.

For *I*_1_(*q*) the maximum probability was calculated using a scaling factor *c*_1_ of 10000 and a *D*_*max*_ of 198 nm. The reconstructed curve 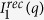 is displayed in Fig. 3(a) and the corresponding PDDF 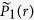 is shown in Fig. 3(b). In this case we observe that the distribution 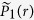 is a continuous curve with one distinct maximum. Such a profile was also observed in Fig. 2 and indicates that for neutrons *ρ*_*shell*_ > *ρ*_*sphere*_ > *ρ*_*solvent*_. The maximum of 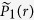 is at *r* ≈ 70 nm, which correlates for spherical particles with a homogeneous scattering length density profile to a mean diameter of 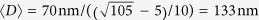. This is in quite good agreement with AF4-MALS were the weight-based mean diameter of the multi-core particles was estimated to be 〈*D*〉 ≤ 144 nm. Indeed, when we compare the shape of 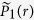 to the equivalent simulated distribution in Fig. 2 we see that it is considerably more asymmetric at large values of *r*. This indicates a moderately broad size distribution with a maximal size of 198 nm, which is in excellent agreement with our observation via AF4-MALS (Fig. 1(c)). We thus conclude that the PDDF determined via numerical inversion of SANS data is a good indication of the size distribution of the individual multi-core particles.

If we now consider X-ray scattering data, we obtain the highest evidence of the combined data *I*_2_(*q*) with a *c*_2_ of 10964.78 and a *D*_*max*_ of 166 nm. The reconstructed curve 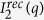 is displayed in Fig. 3(a) and the corresponding PDDF 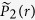 is shown in Fig. 3(b). The PDDF 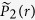 significantly deviates from 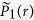 and exhibits two distinct maxima, with two pronounced zero crossings (Fig. 3(b)). Again, this is qualitatively identical to the simulated distribution in Fig. 2, which was determined for *ρ*_*shell*_ > *ρ*_*solvent*_ > *ρ*_*sphere*_. Due to the zero crossing of 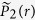 with the minima at about *r* = 80 nm, the reconstructed scattering intensity 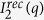 (Fig. 3(a)) has a negative slope in the region *q* ~ *π*/*r* = 0.04 nm^−1^. Similar scattering behaviour and PDDFs have previously been observed in the literature for small micelles^43^.

By combining the inversion of the SLS and SANS or SAXS data, we yield additional information about the scattering behaviour of the particles in the low *q*-range. However, the SLS data is not necessarily identical to the expected data for neutrons or X-rays due to the different SLD contrasts for light, neutrons and X-rays. In order to verify that iron oxide cores are distributed in the surface region of the spheres, we limited our data analysis to the SANS and SAXS data, with *D*_*max*_ as the only free fit parameter (*D*_*max*_ = 100–500 nm, 201 steps).

The obtained PDDFs are also shown in Fig. 3(b). For the SANS data we see that the pair distance distribution function 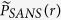 is qualitatively identical to that obtained with inclusion of the SLS data (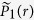). For SAXS only data, the *q*-range had to be limited to 1.8 nm^−1^, because of the high point density and noise for *q* < 1.8 nm^−1^. Even with the limited *q*-range, we find the highest evidence when *D*_*max*_ is 166 nm, which is is identical to that determined from the inversion of the complete data set *I*_2_(*q*). Also, 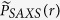 exhibits the two characteristic peaks associated with particle structure, which confirms the previous results. Hence it can be concluded from the combined analysis of *I*_*SLS*_(*q*), *I*_*SANS*_(*q*) and *I*_*SAXS*_(*q*), that the iron oxide nanoparticle cores seem to be mostly embedded in the outer surface layers of the poly(styrene) spheres resulting in the structure illustrated in Fig. 1(a) (*Inset 3*).

## Magnetic properties

### Theory

According to TEM and XRD the small cores are spherically shaped and have a mean diameter in the range 7–9 nm. Thus, they can be expected to be single-domain and superparamagnetic particles^59^ with macrospins 
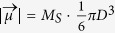
, where *M*_*S*_ is the material specific saturation magnetisation^18^,^19^.

Neglecting anisotropy and dipolar interactions between the cores, the isothermal magnetisation of a monodisperse, superparamagnetic spin ensemble can be described by a single Langevin-function^60^


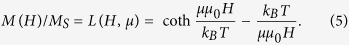


Within polydisperse ensembles, however, there is a distribution of sizes and consequently the magnitude of moments is also distributed. In this case, *M(H*) is modeled by:


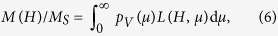


where *p*_*V*_(*μ*) is the volume weighted moment distribution. As core diameters tend to be log-normally distributed, the standard approach is to model magnetisation via equation 6 assuming a log-normal distribution of the moments^22^,^23^,^24^. However, literature contains several different approaches^35^,^36^,^37^ to extract discrete moment distributions with no a priori assumptions *how* the moments are distributed. In this study we apply the same numerical approach for the determination of the moment distribution *p(μ*) from the magnetisation data as for the indirect Fourier transform. The numerical details are provided in the Methods section.

### Data analysis

**Figure 4:**
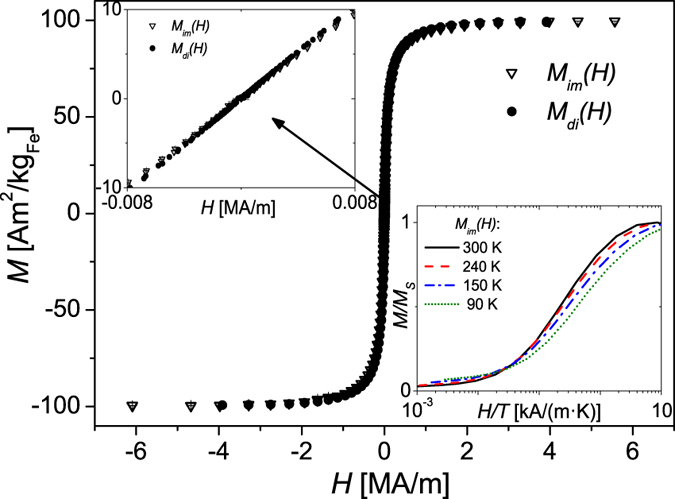
Isothermal magnetisation curves of the immobilised particles (*M*_*im*_(*H*)) and of the colloidal dispersion (*M*_*di*_(*H*)) at *T* = 300 *K*. *Inset top*: Zoom into low-field range. *Inset bottom*: Temperature dependent magnetisation curves of immobilised particles as function of *H*/*T* (log-normal scale).

We confirm that the cores are indeed superparamagnetic at 300 K (i.e. thermally unblocked) by the curves in Fig. 4. Both the magnetisation curve of the immobilised particles *M*_*im*_(*H*) as well as of the colloidal dispersion *M*_*di*_(*H*) are anhysteretic and exhibit zero remanence and coercivity. However, the measured saturation magnetisation *M*_*S*_ ≈ 101 Am^2^/kg_Fe_ of the particles is below the literature values for magnetite (128 Am^2^/kg_Fe_) and maghemite (118 Am^2^/kg_Fe_)^18^. It is typical to observe a reduction in *M*_*S*_ of nanoparticulate material because of structural defects leading to uncorrelated surface spins^61^,^62^.

**Figure 5:**
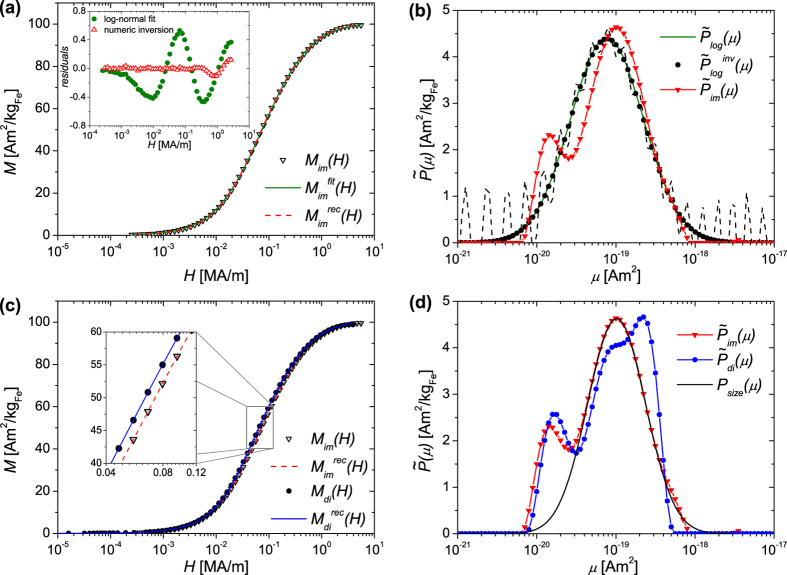
Magnetic characterisation of the multi-core particles at *T* = 300 *K*. (a) Initial magnetisation curve of *M*_*im*_(*H*) from Fig. 4, fit 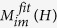 with equation 6 under assumption of a log-normal distribution *p*_*V*_(*μ*) and reconstructed data set 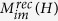 (numerical inversion). *Inset*: Residuals of the log-normal fit and of the numerical inversion. (b) Log-normal distribution of magnetic moments 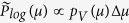 determined by fitting *M*_*im*_(*H*) with equation 6. The fitting curve 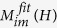 with artificially added noise was numerically inversed, resulting in the distribution 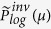 (dashed line shows the extracted distribution in case of a reduced regularisation parameter). With the same numerical approach the distribution 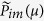 was extracted from the experimental data *M*_*im*_(*H*). (c) Initial magnetisation curves of *M*_*di*_(*H*) and *M*_*im*_(*H*) from Fig. 4 and the reconstructed data sets 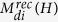 and 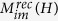. For the reconstruction the moment distributions 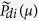 and 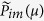 from Fig. 5(d) were used. (d) Discrete moment distributions 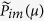 (from Fig. 5(b)) and 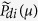 determined by numerical inversion of the initial magnetisation curves of *M*_*im*_(*H*) and *M*_*di*_(*H*). The distribution *P*_*size*_(*μ*) was calculated for a particle ensemble with a mean core size from a log-normal distribution of 6.6 nm (*σ* = 0.28).

In order to extract the intrinsic distribution of the core moments, first we have used equation 6 to fit the virgin curve of the magnetisation curve measured on the immobilised particles, *M*_*im*_(*H*), under assumption of a log-normal distribution *p*_*V*_(*μ*). The fitting curve 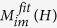 is shown in Fig. 5(a) and the corresponding distribution 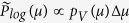 is displayed in Fig. 5(b). In doing this, we obtain a mean magnetic moment of 1.3 ⋅ 10^−19^ Am^2^ with *σ* = 1.06. Using the measured saturation magnetisation the distribution *p*_*V*_(*μ*) is transferred to a number-weighted size distribution *p*_*N*_(*D*) with the mean value 5.4 nm and the broadness *σ* = 0.35. The residuals show systematic deviations over the whole field range (Fig. 5(a)) and illustrate the limitations of using a predetermined moment distribution, a topic which has been discussed in^35^,^36^,^37^.

Therefore, in the following we apply the numerical inversion approach introduced in the Methods section to extract the discrete, apparent moment distribution. First we try to validate the approach. For this purpose, we numerically inversed the fitting curve 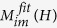 from Fig. 5(a) to which we artificially added reasonable noise and errors. In fact, we used as standard deviations the experimental error bars Δ*M(H*) of the measured curve *M*_*im*_(*H*) and shifted the data points randomly either by +Δ*M(H*) or by −Δ*M(H*). The data set was numerically inversed for 200 different *α* values and afterwards the evidence was calculated for each regularisation parameter *α*. In this case, the highest evidence was obtained for *α* = 3007 and the corresponding distribution 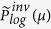 is plotted in Fig. 5(b). Comparison of 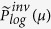 with the *intrinsic* moment distribution 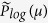 shows that they are identical and hence validates our approach. For comparison, when *α* is reduced to for example 0.01 the extracted distribution exhibits artificial and systematic oscillations over the whole *μ*-range (Fig. 5(b), dashed line).

We now use the numerical inversion approach to analyze the experimental data. Figure 5(b) shows the discrete, apparent moment distribution 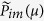 obtained by a numerical inversion of *M*_*im*_(*H*) of the immobilised particles. A reconstruction of *M(H*) with 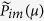 results in 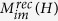 plotted in Fig. 5(c). As shown in Fig. 5(a), the residuals 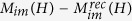 are significantly reduced over the whole field range compared to the log-normal fit, indicating the high quality of the fit by numerical inversion. The main feature of the extracted moment distribution 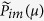 is that it exhibits two distinct peaks, in contrast to the single log-normal distribution 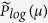. The main peak of 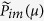 displays the shape of a log-normal peak, which leads us to suggest that this peak is directly correlated to the physical core sizes, which normally are log-normally distributed.

To further examine this, the main peak was modeled to extract the core size distribution from the moment distribution, assuming a log-normal size distribution. The best agreement was found for the distribution *P*_*size*_(*μ*) shown in Fig. 5(d), where 
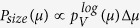
 (Δ*μ* is logarithmically spaced). This distribution was calculated for a particle ensemble with a saturation magnetisation of *M*_*S*_ = 101 Am^2^/kg_Fe_ assuming the stoichiometry of maghemite. The number weighted size distribution used for the computation of *P*_*size*_(*μ*) was a log-normal distribution with a mean value of 6.6 nm (*σ* = 0.28). This mean is smaller than the diameters observed by TEM (Fig. 1(a), 〈D〉 = 9.0(2)nm and *σ* = 0.23(2)) although it is comparable to that deduced from XRD (7(1)nm). Indeed, it is typical for the so-called *magnetic* size to be up to 1 nm smaller than the physical size, an observation conventionally attributed to uncorrelated surface spins^61^,^62^,^63^,^64^. Hence, we conclude that the *P*_*size*_(*μ*) distribution provides a good approximation of the intrinsic moment distribution of the individual cores.

The small peak in 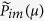 could then be interpreted as a second fraction of small particles with a quite narrow size distribution. The peak position is at 1.4 ⋅ 10^−20^ Am^2^, which corresponds to particle diameters of ≈3 nm. It is important to note that this distribution is volume-weighted; when we convert it to a number-weighted distribution then we would expect to observe the corresponding peak to be about 1 order of magnitude larger than the main peak. This would indicate that the overwhelming majority of cores were ≈3 nm in size, which is not supported by TEM (Fig. 1(a)). A more reasonable explanation is that this peak is instead a signature for dipolar interactions within the particle ensemble, which would hinder the magnetisation of an ensemble as observed both by simulations^31^,^32^,^33^,^34^ and experimentally^29^. As a result, the apparent moments determined from analysing magnetisation curves of interacting nanoparticle ensembles are usually smaller than the real, intrinsic core moments^65^.

A simple approach to investigate in the superparamagnetic state if dipolar interactions are present is to measure the magnetisation curve at different temperatures *T*^66^. In case of a non-interacting superparamagnetic ensemble the magnetisation curves as a function of *H*/*T* should superimpose. For this purpose we measured *M(H*) of the immobilised ensemble at *T* = 300, 240, 150 and 90 K. We observe that the curves *M(H*/*T*) do not superimpose, with deviations particularly in the intermediate to high field range (inset, Fig. 4). Such an observation strongly emphasises the assumption of the presence of weak dipolar interactions between the cores in the multi-core particles. In turn, we thus conclude that the small peak observed in 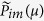 is in fact most likely the result of dipolar interactions.

The presence of two distinct peaks in the moment distribution can then be thought of as a hallmark of a mixture of interactions. The main peak (large moment range) corresponds to the magnetic moments that behave Langevin-like. The second peak in the small moment range corresponds to cores that experience dipolar interactions. For these cores the apparent moment distribution is shifted to smaller values compared to their *real*, intrinsic moment distribution, which is consistent with the mean field approach introduced in ref. 28. In ref. 28, the authors model the magnetisation behaviour of interacting ensembles of magnetic nanoparticles using the Langevin model by introducing an apparent moment *μ*_*a*_, which is related to the real moment *μ* by *μ*_*a*_ = 1/(1 + T^*^/*T*) ⋅ *μ*. Here T^*^ is an effective temperature whose magnitude depends on the average dipolar energy within the ensemble. For small average distances between the particles and hence larger dipolar energies T^*^ ≫ *T* and thus *μ*_*a*_ ≪ *μ*.

This peak in the small moment range is also observed in the distribution 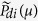 (Fig. 5(d)), which was extracted by numerical inversion of the measurement *M*_*di*_(*H*) of the colloidal dispersion. Another similarity between 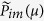 and 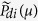 is the position of the main peak, where we observe a shoulder in 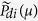. This strongly suggests that both peaks of 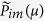 are *real* and correlate directly to the magnetisation behaviour of the iron oxide cores itself. As discussed above, the main peak corresponds to non-interacting cores whereas the small peak can be interpreted as a signature of core-core interactions. However, in contrast to 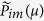, the distribution 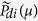 exhibits a third peak at large *μ* values. This peak can be linked to the slightly increased susceptibility of *M*_*di*_(*H*) in the intermediate field range compared to *M*_*im*_(*H*) (Fig. 5(c)). Considering that both samples contained particles from the same synthesis batch, and differ only by the preparation method for the measurement, this observation indicates that some of the multi-core particles had remanent (effective) moments larger than the moments of the individual cores. In colloidal dispersion these multi-core particles could rotate in field direction by physical rotation which increased the susceptibility of the ensemble. A possible explanation for remanent moments within such multi-core particles is that the superparamagnetic spins of the cores are slightly correlated due to dipolar interactions leading to the formation of a blocked effective moment^67^.

## Conclusion

In this work the structural and magnetic properties of multi-core particles were investigated using the same numerical inversion method for the analysis of small angle scattering and magnetisation measurements. The multi-core particles consisted of large poly(styrene) spheres (ca. 160 nm) with embedded superparamagnetic iron oxide nanoparticle cores (ca. 9 nm). By a numerical inversion (indirect Fourier transform) of the SANS and SAXS scattering intensities in combination with the SLS scattering intensity, the PDDFs of the multi-core particles were extracted. Analysis of the PDDFs strongly indicated that the cores were mostly accumulated in the surface layers of the poly(styrene) spheres.

The same numerical approach was applied to analyse the isothermal magnetisation curves of the multi-core particles. First, the apparent, discrete moment distribution of the multi-core particles was extracted from the isothermal magnetisation measurement of the immobilised ensemble. This distribution exhibited two distinct peaks. Whereas the main peak could be correlated to the intrinsic moment distribution of the individual, non-interacting iron oxide nanoparticle cores, the second peak in the low moment range of the apparent moment distribution could be attributed to weak dipolar interactions.

In comparison to the immobilised ensemble, the magnetisation curve of the particles dispersed in water (colloidal dispersion) had a slightly increased susceptibility in the intermediate field range. Consequently, the extracted apparent moment distribution was partially shifted to larger moment values. As a possible explanation for this effect a coupling of the spins of the cores within some of the multi-core particles was proposed, resulting in the formation of finite effective/remanent moments. In colloidal dispersion these particles could rotate in field direction by physical rotation which would explain the increased susceptibility of the ensemble.

The revelation of fine details regarding the structural and magnetic properties of the multi-core nanoparticles proofs the validity and universal applicability of the inversion method as a tool to analyse such novel magnetic nanoparticle arrangements. The code for the numerical inversion of the small angle scattering and isothermal magnetisation data was written in Python and is available from the authors.

## Methods

### Structural characterisation

*Inductively coupled plasma optical emission spectroscopy* (ICP-OES) was carried out with a Thermo Scientific iCAP 6500 ICP emission spectrometer, to determine the iron content in the colloidal dipsersion. For this purpose the colloidal dispersion was digested in 70% HNO_3_ for a week, followed by preparing a dilution series in MiliQ water.

*Transmission electron microscopy* (TEM) images were taken on a JEOL JEM 2100 (FEG) and a FEI Tecnai G2 T20 TEM. The samples were prepared by drop-casting diluted dispersions of the particles on a carbon coated copper grid.

*X*-*ray diffraction* (XRD) patterns were measured with a Bruker D8 Advance diffractometer, using Cu-K*_α_* radiation, with a Bragg-Brentano configuration. The freeze-dried sample was placed on a Si single-crystal low background sample holder that was rotated at 15 rpm in order to minimize the effect of preferred orientations in the sample. The measurements were performed at room-temperature (*T* = 300 K) in the 23°–85° 2Θ range. The instrumental calibration is based on standard Si and LaB_6_ samples. The Rietveld refinement has been performed using the FullProf suite^68^.

*Asymmetric flow field flow fractionation* (AF4) was performed using the instrument Eclipse 2 (Wyatt Technology, Dernbach, Germany) which was connected to a multi-angle light scattering detector (MALS, Dawn Heleos II, Wyatt Technology) operating at a wavelength of 658 nm and to a RI detector (Optilab T-Rex, Wyatt Technology) operating at 658 nm. An Agilent 1100 G1311Aisocratic pump, with an in-line vacuum degasser and auto sampler, delivered the carrier flow and handled sample injection onto the AF4 channel. The AF4 channel (Wyatt Technology) had a tip-to-tip length of 17.4 cm, assembled with a 250 μm spacer and an ultrafiltration membrane of regenerated cellulose (cutoff 10 kDa, Merck Millipore, Billerica, MA, USA). The carrier liquid consisted of MilliQ water with 0.02% NaN_3_. A volume of 30 μl of the diluted colloidal dispersion (concentration 0.2 mg/ml) was injected at 0.20 ml/min for 1 min. The focus time was 5 min at a flow rate of 1 ml/min. An exponential decay cross-flow rate of 1 to 0.15 ml/min, with half-life of 8 min, was applied during elution. At the end of the decay, the cross-flow was held at 0.15 ml/min for 10 min and finally turned off for 10 min to flush the channel. The detector flow was kept constant at 1 ml/min. Data processing was by the Astra software (v.6.1.2.84, Wyatt Technology). The molar mass (*M*) and the gyration radius (*R*_*g*_) were obtained from the light scattering data using the Berry method^69^. The hydrodynamic diameter (*D*_*h*_) was determined based on the retention time^50^.

*Static light scattering* (SLS) was performed using a multi-angle detector set-up equipped with a He-Ne laser by ALV, Langen, Germany. For the measurements the colloidal dispersion was diluted by factors 100, 200, 500, 1000 and 2000 to receive a dilution series. The SLS data were converted to *I(q*) sets, by using the expression *q* = (4*πn*/*λ*)sinΘ with *λ* = 632.8 nm, and normalised so that the first data point *I(q*_*min*_) = 1. All 5 data sets were averaged to obtain *I*_*SLS*_(*q*) but the first data point was excluded due to the fact that it naturally had no standard deviation.

*Small Angle Neutron Scattering* (SANS) of the colloidal dispersion was carried out on the Sans2d small angle diffractometer at the ISIS Pulsed Neutron Source (STFC Rutherford Appleton Laboratory, Didcot, U.K.)^70^. A collimation length of 4 m and incident wavelength range of 1.75–16.5 Å was employed. Data were measured simultaneously on two 1 m^2^ detectors to give a *q*-range of 0.0042–1.45 Å^−1^. The small angle detector was positioned 4 m from the sample and offset vertically 60 mm and sideways 100 mm. The wide-angle detector was position 2.4 m from the sample, offset sideways by 980 mm and rotated to face the sample The beam diameter was 8 mm. Each raw scattering data set was corrected for the detector efficiencies, sample transmission and background scattering and converted to scattering cross-section data using the instrument-specific software MANTID^71^. The *I*_*SANS*_(*q*) data were placed on an absolute scale [cm^−1^] using the scattering from a standard sample (a solid blend of hydrogenous and perdeuterated poly(styrene)).

*Small Angle X*-*ray Scattering* (SAXS) measurements of the colloidal dispersion were carried out on a Kratky system with slit focus, SAXSess by Anton Paar, Graz, Austria. The colloidal dispersion was measured as delivered after vortexing. The measurement was performed as an absolute intensity measurement by measuring additionally the scattering curves of the empty capillary and water. These were subtracted from the measured scattering curve of the sample during the data reduction procedure using SAXSquant software shipped with the machine. The resultant curve *I*_*SAXS*_(*q*) was deconvoluted with the beam profile curve to correct for the slit focus smearing.

### Isothermal magnetisation measurements

The magnetic properties of the composite particles were investigated by analysing the DC magnetisation curves of the immobilised particles (physical rotation of particles suppressed) as well as of the colloidal dispersion (as prepared).

To analyse the magnetic properties of the *immobilised nanoparticles* it was necessary to suppress a rotation of the particles in field direction. This was achieved by depositing a droplet of 5 μl of the colloidal dispersion on cotton wool and letting it dry for 24 hours. The isothermal magnetisation measurement was recorded with a Quantum Design SQUID VSM 7T with Quick Switch and Evercool at 300 K in a field range of *μ*_0_*H* = ±5.6 MA/m. The magnetisation *M*_*im*_(*H*) was obtained by normalising to the iron content, determined by ICP-OES.

Magnetisation measurements were performed on the *colloidal dispersion* at 300 K in a Magnetic Property Measurement System (MPMS)-XL (Quantum Design, USA). The magnetic field was varied between ±3.9 MA/m and the size of consecutive field step was changed logarithmic to ensure a sufficient number of points at low fields. From the measured magnetic moment the magnetic contribution of the empty sample holder and the diamagnetic signal of water were subtracted. The corrected magnetic moment was normalised to the mass of iron, which was determined by ICP-OES, to obtain the magnetisation *M*_*di*_(*H*).

### Numerical inversion

#### Small angle scattering data (Indirect Fourier transform)

The measured scattering intensity *I(q*) is a vector with *M* data points. At each data point *I(q*_*i*_) the integral equation 1 can be written in the discrete form


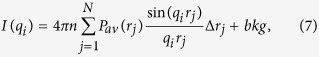


with *n* being the number density of the particles and *P*_*av*_(*r*) their averaged PDDF. The histogram *P*_*av*_(*r*) is divided in *N* bins with width Δ*r*_*j*_. To extract the *N*-dimensional vector 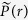 with 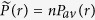 the functional


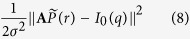


has to be minimised, with *σ* = *σ(q*) being the standard deviation of each data point. Here *I*_0_(*q*) is the measured scattering intensity *I(q*) minus the incoherent scattering background (*I*_0_(*q*_*i*_) = *I(q*_*i*_) − *bkg*). The matrix **A** in equation 8 is the *M* × *N* data transfer matrix with 
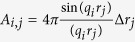
. Due to e.g. measurement uncertainties solving equation 8 is normally an ill-conditioned problem, which can give rise to unphysical peaks in the determined histogram. To make the problem less ill-conditioned a Tikhonov regularisation was applied to force smooth distributions. In this case the functional^72^


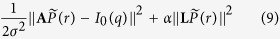


is minimised instead of equation 8. The matrix **L** is a *N* × *N* regularisation matrix, which is weighted by the regularisation parameter *α*. To penalise oscillations within the extracted distributions and to force 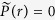 at the start and end point, the following non-singular approximation of the discrete second-order derivative operator was used within this work:


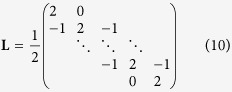


This operator is basically identical to the regularisation functional used in ref. 39 and is similar to Glatter’s original smoothness constraint^40^. For numerical computation equation 9 is inconvenient and the least square solution of


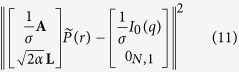


was determined. Here 0_*N*,1_ is a zero vector of length *N*.

The find the optimal value for the regularisation parameter *α*, the posterior probability or evidence *P(α*) was calculated according to^38^,^39^


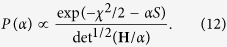


Equation 12 was calculated in refs 38 and 39 within the Bayesian framework and proven to result in correct estimations for the regularisation parameter and hence the extracted pair distance distribution functions. Here *χ*^2^ is defined in the usual manner, i.e.


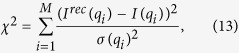


with *I*^*rec*^(*q*_*i*_) being the reconstructed data points:


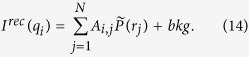


The functional *S* in equation 12 is


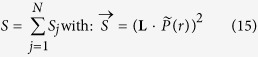


and **H** is the Hessian of the Tikhonov functional (equation 9):


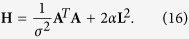


In this work the IFT was used to determine the PDDFs of the particles by inversing the SANS scattering intensity *I*_*SANS*_(*q*), the SAXS scattering intensity *I*_*SAXS*_(*q*) or combinations of *I*_*SANS*_(*q*) and *I*_*SAXS*_(*q*) with the SLS scattering intensity *I*_*SLS*_ (*I*_1_(*q*) = (*I*_*SLS*_(*q*), *I*_*SANS*_(*q*)), *I*_2_(*q*) = (*I*_*SLS*_(*q*), *I*_*SAXS*_(*q*))). For this purpose the *bkg*-values had to be subtracted from the scattering intensity (equation 11, *I*_0_(*q*_*i*_) = *I(q*_*i*_) − *bkg*). In principal *bkg* can be included as a fit parameter as done in ref. 39. However in this work the values were pre-determined by a linear fit of the scattering data in the high *q*-range to *bkg*_*SANS*_ = 0.0182 cm^−1^ and *bkg*_*SAXS*_ = 0.0602 cm ^– 1^ and subtracted before the IFT.

Another prerequesite for the IFT is that the histogram 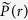 is restricted to the range 0 < *r* ≤ *D*_*max*_, with *D*_*max*_ being the maximum size of the largest scatterers within the ensemble^39^. According to AF4-MALS, *D*_*max*_ of the individual multi-core particles can be roughly estimated to be *D*_*max*_ ≤ 253 nm. Additionally some agglomerates of the multi-core particles were observed with maximum *R*_*g*_ values up to 180 nm (Fig. 1(c)). To certainly find the optimal value for *D*_*max*_ for the IFT, the IFT was performed for a total of 201 *D*_*max*_ values ranging from 100 to 500 nm in 2 nm steps. For each *D*_*max*_ value the distribution 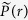 was set to be from *r*_*j*_ > 0 → *D*_*max*_, divided in *N* = 500 bins with a linear spacing. Furthermore, at each *D*_*max*_ value the regularisation parameter *α* was varied over several orders of magnitude in 70 steps with logarithmic spacing. After each IFT the evidence *P(α, D*_*max*_) = *P(α*) was computed with equation 12 to find the most probable values for *α* and *D*_*max*_ (highest evidence). The distribution 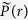 determined by the IFT for these particular values of *α* and *D*_*max*_ was regarded as the most probable solution for the PDDF.

However, for the inversion of the combined data sets *I*_1_(*q*) and *I*_2_(*q*), additionally the absolute values of *I*_*SLS*_(*q*) in relation to *I*_*SAXS*_(*q*) and *I*_*SANS*_(*q*) were not known. Hence, the scaling factors *c*_1_ and *c*_2_ of the intensity *I*_*SLS*_(*q*) were introduced as third fit parameters with *I*_1_(*q*) = (*c*_1_ ⋅ *I*_*SLS*_(*q*), *I*_*SANS*_(*q*)) and *I*_2_(*q*) = (*c*_2_ ⋅ *I*_*SLS*_(*q*), *I*_*SAXS*_(*q*)). These scaling factors were varied in both cases over two orders of magnitude from 10^3^ to 10^5^ in 51 steps with logarithmic spacing. Finally, for a given set of *α, D*_*max*_ and *c*_1,2_ the IFT was performed and afterwards the corresponding probabilities *P(α, D*_*max*_, *c*_1,2_) = *P(α*) calculated with equation 12. The distribution 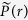 determined with the parameters for which the highest evidence was calculated was then interpreted as the best estimation for the PDDF.

#### Isothermal magnetisation data

Identically to the scattering intensity (equation 7), the magnetisation data is a vector and equation 6 can be discretised as:


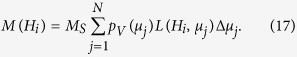


By finding the least square solution of (see equation 11)


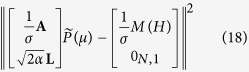


the vector 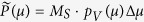 was determined, with *A*_*i,j*_ = *L(H*_*i*_, *μ*_*j*_).

For the inversion the range of the extracted moment distribution was set to be from 10^−21^–10^−15^ Am^2^, divided into *N* = 121 bins (20 points per decade) with a logarithmic spacing Δ*μ*. The inversion was performed for 200 different values of *α*, with *α* being varied logarithmically over several orders of magnitude. Afterwards, the evidence was calculated with equation 12, as for the IFT, and the distribution with the highest evidence interpreted as the most probable solution 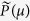.

## Additional Information

**How to cite this article**: Bender, P. *et al*. Structural and magnetic properties of multi-core nanoparticles analysed using a generalised numerical inversion method. *Sci. Rep.* **7**, 45990; doi: 10.1038/srep45990 (2017).

**Publisher's note:** Springer Nature remains neutral with regard to jurisdictional claims in published maps and institutional affiliations.

## Figures and Tables

**Table 1:** SLDs of H_2_O (solvent), poly(styrene) (sphere) and iron oxide for neutrons and X-rays.

	neutrons	X-rays
*ρ*H_2_O = *ρ*_*solvent*_	−5.6 · 10^−5^ 1/nm^2^	9.5 · 10^−4^ 1/nm^2^
*ρ*poly(styrene) = *ρ*_*sphere*_	1.3 · 10^−4^ 1/nm^2^	9.2 · 10^−4^ 1/nm^2^
*ρ*_iron oxide_	6.7 · 10^−4^ 1/nm^2^	40.6 · 10^−4^ 1/nm^2^
*ρ*_*shell*_	1.8 · 10^−4^ 1/nm^2^	9.7 · 10^−4^ 1/nm^2^

As iron oxide magnetite (Fe_3_O_4_) was assumed. For poly(styrene) (chemical formula (C_8_H_8_)_*n*_) the volumetric mass density 1_*g*_/cm^3^ was used for the calculation. The SLDs *ρ*_*shell*_ > *ρ*_*sphere*_ were used for the calculations of the PDDFs of the particles (Fig. 2).
